# Labial adhesions as a rare mucosal manifestation of bullous lupus erythematosus in a 15-year-old female

**DOI:** 10.1097/JW9.0000000000000256

**Published:** 2026-02-11

**Authors:** Glen Aldrix R. Anarna, Aikaterini Patsatsi, Josef Symon S. Concha

**Affiliations:** a Department of Dermatology, University of the Philippines, Philippine General Hospital, Manila, Philippines; b 2nd Dermatology Department, Aristotle University School of Medicine, Papageorgiou General Hospital, Thessaloniki, Greece

**Keywords:** bullous lupus erythematosus, cutaneous lupus erythematosus, genital lupus, labial adhesion, systemic lupus erythematosus

**What is known about this subject in regard to women and their families?**

- Lupus erythematosus (LE) is an autoimmune disease commonly affecting women of reproductive age with diverse clinical manifestations, affecting the skin or multiple systems. Cutaneous symptoms appear in 70% of systemic LE cases, but vesiculobullous lesions occur in less than 5%. Mucosal involvement typically includes oral, nasopharyngeal, and ocular ulcerations, while genital lupus remains rare.

**What is new from this article as messages for women and their families?**

- We report a Filipino adolescent with recurrent vesiculobullous skin lesions, painless oral ulcerations, and vaginal ulcerations leading to labial adhesions, dysuria, and recurrent urinary tract infections. This case highlights labial adhesions as a rare mucosal manifestation of bullous lupus erythematosus and emphasizes the need for multidisciplinary management.

## Dear Editors,

Bullous lupus erythematosus (LE) is a rare cutaneous manifestation of LE, with a few cases described in childhood.^1^ While mucosal involvement usually includes oral or nasopharyngeal ulcerations, ocular manifestations, and serositis, genital lupus has rarely been reported.

**Fig. 1.**
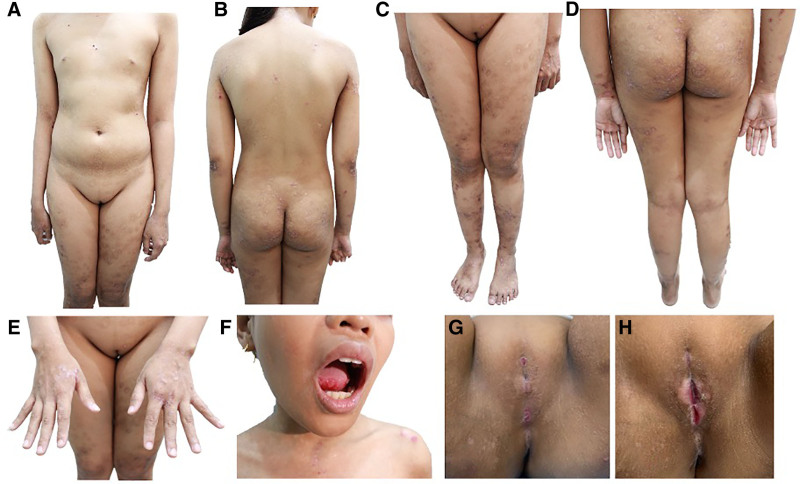
Focused dermatologic examination of the patient showing: (A-D) multiple, well-defined, irregularly shaped, erythematous, erosions with hemorrhagic crusting and excoriations, generalized all over the body. (E) Hypopigmented scarring and milia formation on the dorsal aspect of both hands from previous sites of skin lesions. (F) Oral mucosa findings of multiple, well-defined, round, white, and erythematous plaques on both sides of the tongue. (G) Genital strictures with erythematous borders pre-op (courtesy of Dr. Cecilia Cabanag from the Department of Obstetrics and Gynecology, PGH). (H) Genital lesions 1-month post-op and on 3 months of hydroxychloroquine.

**Fig. 2.**
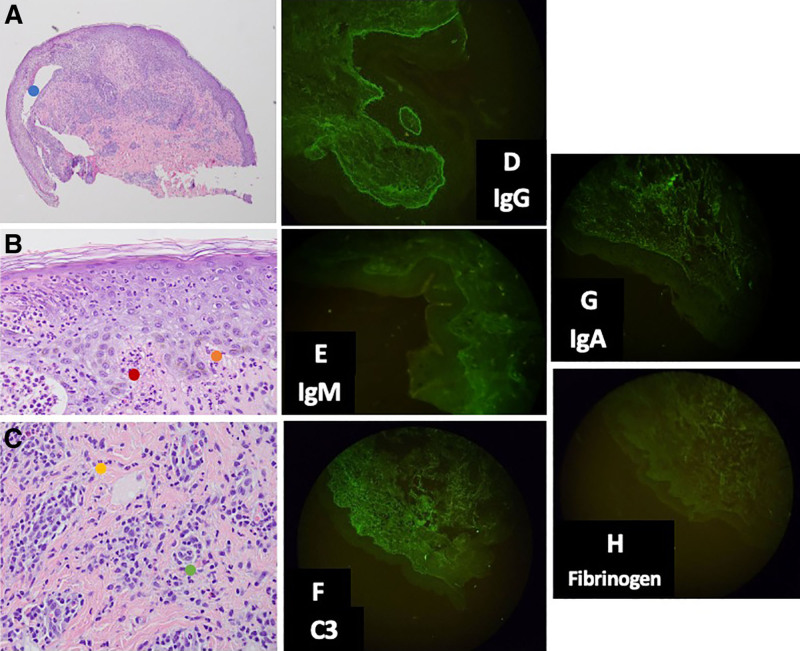
Histopathology with H&E stain: (A and B) subepidermal blister (blue dot) with vacuolar interface change (orange dot). Neutrophils are found along the dermo-epidermal junction with some neutrophilic microabscesses in the papillary dermis (red dot). (C) Dense diffuse infiltrate composed predominantly of neutrophils, located perivascular (green dot) and interstitial (yellow dot) in the superficial to mid-dermis. Immunohistopathology: (D–G) basement membrane zone deposition of IgG (strong deposition), IgM (weak deposition), IgA (weak deposition), and C3 (weak deposition). (H) Negative findings for fibrinogen.

A 15-year-old girl presented with a 5-year history of intensely pruritic, erythematous vesicles and bullae that erode leading to erosions with milia formation. She developed vaginal ulcerations with erythematous borders, causing labial fusion and difficulty in urination (Fig. 1**).** Biopsy of the blister showed a subepidermal split with a vacuolar interface change and dense diffuse neutrophilic infiltrates. Direct immunofluorescence revealed basement membrane zone deposition of IgG, IgM, and IgA (Fig. 2). She had positive antinuclear antibody 1:80, but normal anti-dsDNA and C3 levels. Hormonal tests indicated hypogonadotropic hypogonadism, and imaging showed an infantile uterus with silent ovaries. Complete blood count showed anemia for age with direct Coomb’s test positive (+1). Urinalysis showed albuminuria, hematuria, and positive leukocytes, which may indicate possible lupus nephritis or urinary tract infection. Urine protein-creatinine ratio indicated microalbuminuria. The final assessment was bullous LE and early systemic LE. Epidermolysis bullosa acquisita was considered but excluded, given the vacuolar interface changes favoring bullous LE. Toxic epidermal necrolysis-like acute cutaneous LE was also a differential, though it typically presents hyperacutely on sun-exposed areas. Initial management was triamcinolone acetonide 0.1% lotion, sunscreen, and wound care. She began hydroxychloroquine 200 mg/day and later underwent Z-plasty labiaplasty under spinal anesthesia. Colchicine was added afterwards, with no stricture recurrence.

Labial adhesions and strictures are rare in LE, with only a few documented cases of genital involvement. Reports have described different manifestations, such as atrophic hypopigmented plaques looking like discoid LE, ulcerations, and lichen planus-like lesions in both male and female genitalia,^2^ The variation in clinical presentation makes diagnosis challenging and can lead to misdiagnosis as sexually transmitted infection and manifestation of other autoimmune diseases.^3^

The pathophysiology of recurrent aphthous ulcerations in the context of immune dysregulation remains largely unknown.^4^ Histology showed features typical of LE: hyperkeratosis, perivascular/periadnexal inflammation, basal vacuolar change, apoptotic keratinocytes, and mucin deposition, though not all were consistently present. T-cell activation by mast cells and macrophages and the release of tumor necrosis factor-alpha likely drives the inflammation. Tumor necrosis factor-alpha promotes leukocyte adhesion to endothelial cells and neutrophil chemotaxis, which results in inflammation.^3^ Lack of appropriate treatment can lead to scarring and disfigurement.

Common treatment options include dapsone, superpotent corticosteroids, antimalarial drugs, immunosuppressants, and biologics.^4^ Mycophenolate mofetil has been used to treat autoimmune blistering diseases affecting the mucosal surfaces.^5^ Dapsone is highly effective for bullous LE, but it is not widely available in the Philippines. Surgical intervention should be deferred until medical stabilization to avoid further mucocutaneous damage, as in our patient. Given the risk of severe complications, such as recurrent urinary tract infections and kidney failure, a multidisciplinary approach combining medical and surgical treatments is recommended for managing genital LE and its complications.

## Conflicts of interest

None.

## Funding

None.

## Study approval

N/A.

## Author contributions

GAA: Treating physician and participated in the writing, editing, and review of this manuscript. AP: participated in editing and review of this manuscript. JSC: participated in the writing, editing, and review of this manuscript. All authors reviewed and approved the final manuscript.

## Patient consent

The authors obtained written consent from the patient’s parents for her photographs and medical information to be published in print and online, and with the understanding that this information may be publicly available. Patient consent forms were also provided to the journal and retained by the authors.

## Acknowledgments

The authors thank Dr. Cecilia Cabanag, Department of Obstetrics and Gynecology, Philippine General Hospital.

## References

[1] Ibarra DM Parra AR Ramirez MP. Clinical presentation of bullous systemic lupus erythematosus in a pediatric patient: a case report. Int J Res Dermatol 2023;9:297–9. doi: 10.18203/issn.2455-4529.intjresdermatol20232547.

[2] Romiti R Anzai A Nico M. Genital discoid lupus: a rare manifestation of cutaneous lupus erythematosus. Lupus 2014;23:707–10. doi: 10.1177/0961203314522336. 24548969 10.1177/0961203314522336

[3] Kakani P Zara J. Aphthous genital ulcers: an uncommon manifestation of new-onset systemic lupus erythematosus (SLE). Proc UCLA Health 2022;26. Available from: https://escholarship.org/uc/item/9rx1w20f. Accessed December 7, 2025.

[4] Wang G Wang C Xu X Jia H. Two cases of bullous systemic lupus erythematosus treated successfully with T2 and low-dose corticosteroids. Dermatol Sin 2015;34:92–5. doi: 10.1016/j.dsi.2015.08.004.

[5] Meurer M. Immunosuppressive therapy for autoimmune bullous diseases. Clin Dermatol 2012;30:78–83. doi: 10.1016/j.clindermatol.2011.03.013. 22137230 10.1016/j.clindermatol.2011.03.013

